# Stable Zinc Anodes Enabled by Zincophilic Cu Nanowire Networks

**DOI:** 10.1007/s40820-021-00783-4

**Published:** 2021-12-23

**Authors:** Shiyin Xie, Yang Li, Xu Li, Yujun Zhou, Ziqi Dang, Jianhua Rong, Liubing Dong

**Affiliations:** College of Chemistry and Materials Science, Jinan University, Guangzhou, 511443 People’s Republic of China

**Keywords:** Zn-based energy storage, Zinc anodes, Zinc dendrite, Zincophilic materials, Cu nanowire networks

## Abstract

Zn-based electrochemical energy storage (EES) systems have received tremendous attention in recent years, but their zinc anodes are seriously plagued by the issues of zinc dendrite and side reactions (e.g., corrosion and hydrogen evolution). Herein, we report a novel strategy of employing zincophilic Cu nanowire networks to stabilize zinc anodes from multiple aspects. According to experimental results, COMSOL simulation and density functional theory calculations, the Cu nanowire networks covering on zinc anode surface not only homogenize the surface electric field and Zn^2+^ concentration field, but also inhibit side reactions through their hydrophobic feature. Meanwhile, facets and edge sites of the Cu nanowires, especially the latter ones, are revealed to be highly zincophilic to induce uniform zinc nucleation/deposition. Consequently, the Cu nanowire networks-protected zinc anodes exhibit an ultralong cycle life of over 2800 h and also can continuously operate for hundreds of hours even at very large charge/discharge currents and areal capacities (e.g., 10 mA cm^−2^ and 5 mAh cm^−2^), remarkably superior to bare zinc anodes and most of currently reported zinc anodes, thereby enabling Zn-based EES devices to possess high capacity, 16,000-cycle lifespan and rapid charge/discharge ability. This work provides new thoughts to realize long-life and high-rate zinc anodes.

**Supplementary Information:**

The online version contains supplementary material available at 10.1007/s40820-021-00783-4.

## Highlights

- A novel strategy of employing Cu nanowire networks to realize superior long-life and high-rate zinc anodes was proposed.
- Cu nanowire networks could stabilize zinc anodes from multiple aspects, including homogenizing surface electric field and Zn^2+^ concentration field, inhibiting side reactions and inducing uniform zinc nucleation/deposition.
- Facets and edge sites of Cu nanowires, especially the latter ones, were revealed to be highly zincophilic.

**Supplementary Information:**

The online version contains supplementary material available at 10.1007/s40820-021-00783-4.

## Graphical abstract

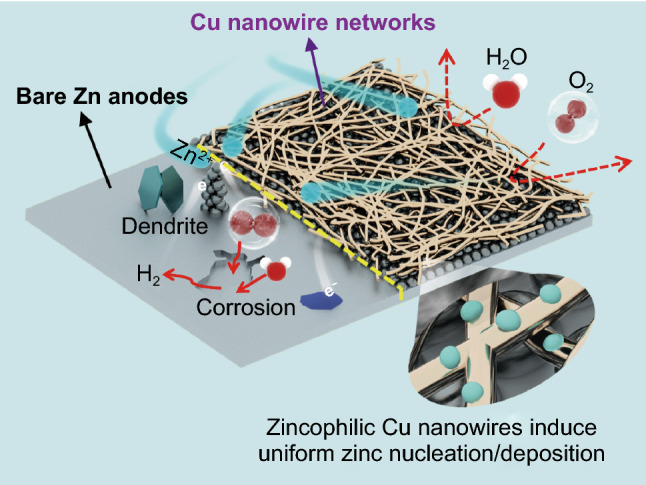

## Introduction

The rapid development of electric vehicles, wearable electronic products and smart grids has aroused enthusiasm for seeking safe, high-energy, low-cost and environmentally friendly electrochemical energy storage (EES) systems. Among various EES systems, aqueous Zn-based EES systems using neutral or mildly acidic electrolytes, typified by zinc-ion batteries and zinc-ion hybrid supercapacitors, have received tremendous attention in recent years [1– 5]. Application of aqueous electrolytes with high ionic conductivity endows the Zn-based EES systems with high safety, low toxicity and the potential for realizing fast charge/discharge. Meanwhile, metallic zinc anodes in the Zn-based EES systems are characterized by high theoretical capacity (820 mAh g^−1^ and 5,845 Ah L^−1^) and relatively low electrochemical potential (− 0.762 V vs. standard hydrogen potential), being beneficial for the Zn-based EES systems to achieve high energy density [6–8]. Moreover, diverse cathode materials such as manganese oxides, vanadium oxides, carbon materials, Prussian blue analogs and organic materials have been explored for aqueous Zn-based EES [9–17], and encouragingly, electrochemical performance of cathode materials for aqueous Zn-based EES devices is continuously improved.

Despite the above merits of aqueous Zn-based EES systems, their practical application is hampered by the electrochemical and thermodynamic instability of metallic zinc anodes [18–20]. To be specific, during zinc plating/stripping processes, the microscopic surface of zinc anodes changes continuously, thereby causing an inhomogeneous electric field (e.g., protuberance sites generally create a strong localized electrical field due to the “tip effect”) [21]. The inhomogeneous surface electric field, coupling with rampant 2D diffusion of Zn^2+^ on the zinc anode surface, raises the issues of zinc dendrites and short circuits [21, 22]. At large charge/discharge currents, these issues become more serious [23]. Meanwhile, the mildly acidic feature and dissolved oxygen of aqueous zinc salt electrolytes such as ZnSO_4_ solutions, as well as relatively high hydrogen evolution potential (vs. Zn^2+^/Zn redox potential), often result in corrosion of metallic zinc and hydrogen evolution reactions, accelerating the failure of zinc anodes [24, 25]. Therefore, aqueous Zn-based EES devices suffer from low coulombic efficiency, unsatisfactory rate performance and inferior cycle lifetime.

To stabilize zinc anodes, several strategies have been proposed such as anode structure design and anode surface modification. For instance, employing a 3D conductive skeleton as the current collector for zinc anodes is beneficial for reducing local current density and thus alleviating the serious aggregation of electrons and Zn^2+^ in particular positions [26, 27]. Artificial interface layers (e.g., porous inorganic particle coating, metal–organic framework layer or polymer coating) introduced on zinc anode surface are capable of guiding Zn^2+^ flux to guarantee uniform deposition of zinc, as well as blocking free water with dissolved oxygen to avoid corrosion of zinc anodes [22, 28–32], but the huge impedance of interfaces between these nonconductive artificial layers and zinc anodes is against rate capability of Zn-based EES devices [33]. Furthermore, when conductive and porous carbon networks are utilized as the artificial interface layers, they may synchronously regulate Zn^2+^ flux and maintain a stable electric field on zinc anode surface to optimize zinc plating/stripping behaviors [34, 35]. Besides the above strategies, very recent research studies found that zinc plating on specific substrates shows low nucleation barriers and fast kinetics [36–40]. In such cases, Zn^2+^ ions are easy to be captured by zincophilic sites on these substrates and react with electrons to form zinc atoms/clusters, and in return, the number of electrons and Zn^2+^ ions accumulated at anode/electrolyte interfaces is reduced, thereby effectively mitigating zinc dendrite growth. This provides a new approach for stabilizing zinc anodes, but only very limited types of zincophilic materials have been investigated, and meanwhile, some zincophilic materials such as N-doped carbon may catalyze water decomposition which is undesired [41]. In addition, the zinc plating process is composed of several successive steps such as electrostatic adsorption of Zn^2+^ and zinc nucleation. However, many of the current strategies mainly focus on modulating one of the above steps to stabilize zinc anodes. For instance, porous inorganic coating on zinc anodes can guide Zn^2+^ flux [32], but hardly induces zinc nucleation; using Cu plates/foams as current collectors can reduce nucleation barriers of zinc deposition [26], but cannot effectively reduce the local current density due to their limited specific surface area [42, 43]. In theory, a comprehensive strategy that can positively affect both Zn^2+^/electron concentration distribution and zinc nucleation is expected to endow zinc anodes with better electrochemical performance.

Herein, we report a strategy of employing zincophilic Cu nanowire networks to stabilize zinc anodes from multiple aspects. The Cu nanowire networks-protected zinc anodes exhibit stable plating/stripping behaviors at various charge/discharge currents and areal capacities, significantly superior to bare zinc anodes and most of currently reported zinc anodes. According to experimental results and COMSOL simulation, for the Cu nanowire networks covering on zinc anode surface, their porous nature favors uniform Zn^2+^ flux, and meanwhile, nanoscale size endows the Cu nanowires with large specific surface area, which helps to reduce local current density and homogenize Zn^2+^ concentration field, thereby restraining zinc dendrite growth. Besides, by virtue of hydrophobic feature, the Cu nanowire networks suppress the direct contact between free water (with dissolved oxygen) and zinc anodes, which inhibits side reactions such as anode corrosion and hydrogen evolution. Density functional theory (DFT) calculations reveal that the facets and edge sites of the Cu nanowires, especially the latter ones, are highly zincophilic to induce uniform nucleation/deposition of zinc. Moreover, electrochemical stability of the Cu nanowire networks-protected zinc anodes is further examined in Zn-based EES devices.

## Experimental Section

### Material Preparation

#### Preparation of Cu Nanowire Networks

0.17 g CuCl_2_·2H_2_O and 0.308 g glucose were dissolved in 50 mL deionized water, and then 1.44 g hexadecylamine was slowly added to the above solution to get a homogeneous light blue emulsion after continuous magnetic stirring for 12 h. The emulsion was transferred to a 100-mL Teflon-lined autoclave and heated at 120 °C for 6 h. After cooling down to room temperature naturally, precipitation products were washed with hexane/ethanol mixture solution (2: 1 in volume) five times through the centrifugation method, followed by washing with deionized water and freeze-drying to obtain Cu nanowire powder. 5 mg of Cu nanowire powder was dispersed in 5 mL of anhydrous ethanol by the ultrasonic dispersion method, and 50 mL of deionized water was added to get a uniform suspension. The suspension was vacuum-filtered through a hydrophilic filter membrane with a pore size of 0.8 μm to fabricate Cu nanowires-coated membranes (denoted as “CuNW/membranes”), in which Cu nanowires (0.4 mg cm^−2^) randomly dispersed on the membrane to form Cu nanowire networks. The CuNW/membranes were cut into disks with a diameter of 10 mm.

#### Synthesis of Hydrous Ruthenium Oxide/Graphene (GR) Nanocomposite Cathode Material

5.24 mL of graphene oxide aqueous gel (1.24 wt%, produced by Aladdin Reagent Co. Ltd., China) and 286.6 mg RuCl_3_·3H_2_O were mixed with 20 mL of deionized water under magnetic stirring and transferred to a 50-mL Teflon-lined autoclave, followed by heating at 180 °C for 6 h and naturally cooling down to room temperature. GR nanocomposite was obtained by washing the precipitation products with deionized water and drying overnight at 80 °C.

### Material Characterizations

X-ray diffraction (XRD) tests were performed on a diffraction analyzer (model: Rigaku MiniFlex 600) with Cu Kα (λ = 0.15418 nm) radiation. Micro-morphologies of samples were observed using field emission scanning electron microscopy (SEM) (model: Zeiss Sigma 300) and transmission electron microscopy (TEM) (model: FEI Tecnai G2 F20). Thermogravimetric (TG) measurement was carried out on a simultaneous thermal analyzer (model: TGA2) from 30 to 700 °C with a heating rate of 5 °C min^−1^ under air atmosphere. Water contact angle tests were performed on a contact angle tester (model: DSA-100).

### Electrochemical Measurements

Electrochemical behaviors of zinc anodes with and without the protection of Cu nanowire networks were studied by assembling symmetric CR2032 coin cells, in which non-woven cloth separator and 2 M ZnSO_4_ aqueous electrolyte were utilized. For unprotected zinc anodes (i.e., bare Zn anodes), zinc foil disks were directly used as the electrodes of symmetric cells, while in symmetric cells with Cu nanowire networks-protected zinc anodes, a piece of CuNW/membrane was placed between each zinc foil disk electrode and the separator, and Cu nanowires-coated side of the CuNW/membranes contacted with zinc foils (i.e., the filter membrane substrate of the CuNW/membranes directly contacted with the separator). The zinc foils with a thickness of 70 μm were purchased from Qinghe Haoxuan Metal Materials Co. Ltd., China. The Cu nanowire networks-protected zinc anodes are briefly denoted as “Zn@CuNW” to differentiate them from bare Zn anodes in the following. Rate performance and cycling stability of the symmetric cells were evaluated using galvanostatic charge–discharge (GCD) technique at different current densities (0.1–10 mA cm^−2^) and areal capacities (0.1–10 mAh cm^−2^) on a LAND CT2001A battery testing instrument. Linear polarization and chronoamperometry tests of the bare Zn anodes and Zn@CuNW anodes were performed on an electrochemical workstation (model: CHI760E).

Zinc-ion hybrid supercapacitors were assembled with zinc anodes, GR nanocomposite cathodes and 2 M ZnSO_4_ aqueous electrolyte. To prepare the GR composite cathodes, the GR nanocomposite active material, acetylene black and polyvinylidene fluoride, in a mass ratio of 7:2:1, were mixed with N-methylpyrrolidone and then coated on stainless steel foil, followed by complete drying. Cyclic voltammetry (CV) curves and electrochemical impedance spectroscopy (EIS) spectra (with a frequency range of 0.01 Hz to 100 kHz and an amplitude of 5 mV at open-circuit voltage state) of the zinc-ion hybrid supercapacitors were recorded on the CHI760E electrochemical workstation. GCD tests at current densities of 0.1–1 A g^−1^ were performed on a LAND CT2001A battery testing instrument, while those at 2–20 A g^−1^ were carried out on the CHI760E electrochemical workstation (with higher precision to guarantee the accuracy of testing data).

### Theoretical Calculation and Simulation

COMSOL Multiphysics finite element method was used to theoretically simulate electric field and Zn^2+^ concentration distribution on zinc anode surface during zinc plating processes. Spin-polarized DFT calculations were employed to investigate the adsorption energy of single zinc atom on various substrates, e.g., different facets and edge sites of Cu nanowires. Detailed procedures of the COMSOL simulation and DFT calculations are provided in Supporting Information.

## Results and Discussion

### Material Characterizations

**Fig. 1:**
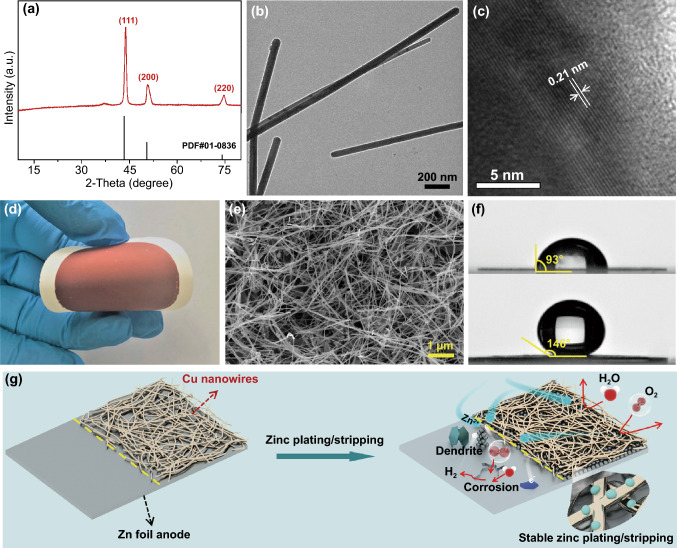
**a** XRD pattern, **b** TEM and **c** high-resolution TEM images of the Cu nanowire sample. **d** Digital photograph and **e** surface SEM image of the CuNW/membrane. **f** Water contact angle of the CuNW/membrane (upper) and Zn foils (below). **g** Schematic illustration of protective effects of Cu nanowire networks on zinc anodes

Physicochemical characteristics of the synthesized Cu nanowire sample are shown in Fig. 1. In the XRD pattern (Fig. 1a), the three diffraction peaks at 2*θ* = 43.3°, 50.4° and 74.2° are indexed to the (111), (200) and (220) crystal planes of face-centered cubic metallic Cu (JCPDS #04–0836). TEM observation shows that most Cu nanowires have a diameter of 40–60 nm and a length of several micrometers (Fig. 1b). Selected area electron diffraction reveals single-crystalline nature of the Cu nanowires (Fig. S1). High-resolution TEM image in Fig. 1c displays interplanar spacing of 0.21 nm that is identified as the (111) crystal plane of the Cu nanowires. An aqueous suspension of the Cu nanowires was vacuum-filtered through a hydrophilic filter membrane, and then Cu nanowire-coated membranes (denoted as “CuNW/membrane”) were obtained (Fig. 1d). For the CuNW/membranes, Cu nanowires disorderly disperse on the surface of the filter membrane to form networks whose thickness is ~ 18 μm, as exhibited by SEM images in Figs. 1e and S2-S3. The Cu nanowire networks are highly hydrophobic, with a water contact angle of 146° (Fig. 1f). By contrast, the water contact angle of bare Zn foils and the filter membrane is only 93° and 34°, respectively (Figs. 1f and S4). This means that when the Cu nanowire networks are used to protect zinc anodes, they are expected to prevent free water and dissolved oxygen inherent in aqueous zinc salt electrolytes from contacting and corroding zinc anodes [22, 44], as illustrated in Fig. 1g.

### Electrochemical Performance

**Fig. 2:**
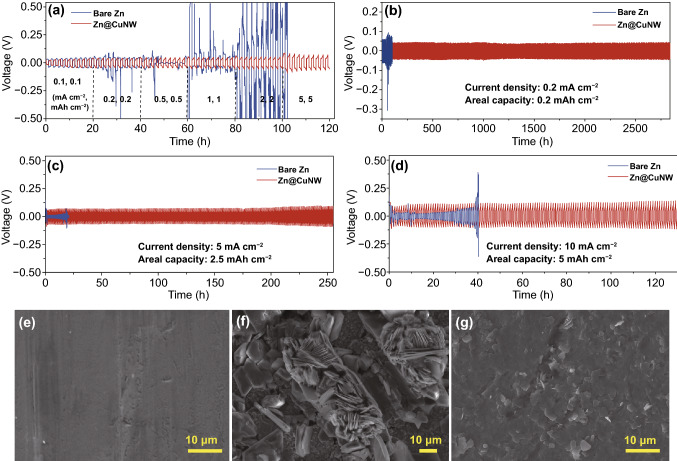
**a** Rate performance of the symmetric cells with bare Zn foil electrodes and Zn@CuNW electrodes. Cycling stability of the symmetric cells at various current densities and areal capacities: **b** 0.2 mA cm^−2^ and 0.2 mAh cm^−2^; **c** 5 mA cm^−2^ and 2.5 mAh cm^−2^; **d** 10 mA cm^−2^ and 5 mAh cm^−2^. SEM images of **e** pristine Zn foil, **f** bare Zn foil after cycling for 100 h, and **g** protected Zn foil after cycling for 100 h

To assess the stabilizing effects of the Cu nanowire networks on zinc anodes, symmetric cells with bare Zn foil electrodes (denoted as “bare Zn”) and the Cu nanowire networks-protected Zn foil electrodes (denoted as “Zn@CuNW”) were assembled (Fig. S5). We can see from Figs. 2a and S6 that Zn@CuNW electrode-based symmetric cells exhibit stable charge/discharge behaviors with small voltage hysteresis at various current densities of 0.1–5 mA cm^−2^ and areal capacities of 0.1–5 mAh cm^−2^. By comparison, bare Zn electrode-based symmetric cells show large voltage hysteresis, and abnormal charge/discharge profiles at relatively large current densities and areal capacities. These prove that zinc plating/stripping on the Zn@CuNW electrodes possesses fast kinetics [32, 45], which is beneficial for realizing high-rate Zn-based EES. Figures 2b and S7 show the long-term cycling stability of these symmetric cells at a current density of 0.2 mA cm^−2^ and an areal capacity of 0.2 mAh cm^−2^. Continuous operation time of Zn@CuNW electrode-based symmetric cells exceeds 2800 h, much longer than that of symmetric cells with bare Zn electrodes (whose operation time is shorter than 100 h), demonstrating that electrochemical stability of zinc anodes can be significantly improved under the protection of the Cu nanowire networks. Moreover, Zn@CuNW//Zn@CuNW symmetric cells display outstanding cycling performance at larger current densities and areal capacities (Figs. 2c-d and S8). Impressively, even at 10 mA cm^−2^ and 5 mAh cm^−2^, Zn@CuNW//Zn@CuNW symmetric cells are capable of operating over 130 h, accompanying with stable charge/discharge behaviors and low voltage hysteresis of only ~ 200 mV (Fig. 2d). Such outstanding cycling stability and rate performance are not only notably superior to those of bare Zn electrode-based symmetric cells, but also have rarely been achieved in previous research studies (Table S1). Note that electrochemical properties of the Cu nanowire networks-protected zinc anodes are affected by the mass loading of the Cu nanowires on the filter membranes (Fig. S9).

Furthermore, since failure of zinc anodes in aqueous zinc salt electrolytes is often caused by the issues of zinc dendrite, corrosion and hydrogen evolution (as illustrated in Fig. 1g) [7], we therefore observed the micro-morphologies of the bare Zn electrodes and the Cu nanowire networks-protected Zn electrodes after repeated zinc plating/stripping processes (i.e., these electrodes-based symmetric cells were charged/discharged for 50 and 100 h at a current density of 5 mA cm^−2^ and areal capacity of 2.5 mAh cm^−2^). As can be seen from Figs. 2e-g and S10, pristine Zn foils have a flat surface, but the bare Zn foil electrodes after cycling exhibit a very bumpy surface covered by many large zinc dendrites/protrusions, and also, some holes and zinc oxide/hydroxide by-products are found (Fig. S10), which can be ascribed to anode corrosion and hydrogen evolution [25, 34]. In sharp contrast, the Cu nanowire networks-protected Zn foil electrodes keep a relatively flat surface without the appearance of large zinc dendrites/protrusions. This confirms again that the Cu nanowire networks effectively protect zinc anodes during zinc plating/stripping processes and improve zinc deposition/stripping efficiency (Fig. S11).

### Mechanism Investigation

**Fig. 3:**
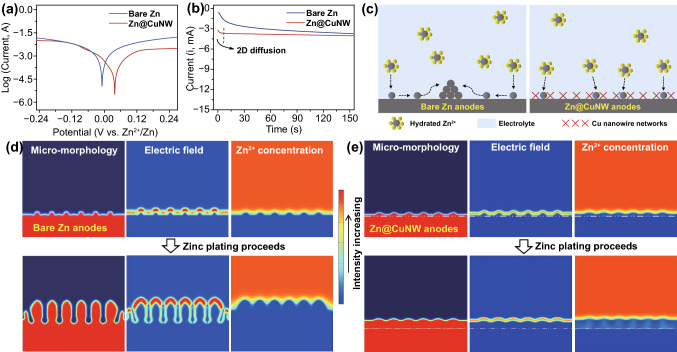
**a** Linear polarization curves and **b** chronoamperometry curves of the bare Zn anodes and Zn@CuNW anodes. **c** Schematic illustration of rampant 2D diffusion behaviors on the bare Zn anodes (left) and restrained 2D diffusion on the Zn@CuNW anodes. Simulation of surface micro-morphology, electric field and Zn^2+^ concentration field during the zinc plating process on **d** bare Zn anodes and **e** Zn@CuNW anodes (the white dash dot lines point out the original position of Cu nanowire networks/zinc anode interface)

The underlying mechanisms of protective effects of the Cu nanowire networks on zinc anodes are investigated in depth. As has been mentioned above, free water and dissolved oxygen-induced corrosion is an important factor causing zinc anode failure. According to linear polarization curves of bare Zn anodes and Cu nanowire networks-protected Zn anodes in ZnSO_4_ aqueous electrolytes (Fig. 3a), the protected Zn anodes show higher corrosion potential and smaller corrosion current, indicating enhanced corrosion-resistant ability [22, 46]. This benefits from the hydrophobic feature of the Cu nanowire networks, which suppresses the direct contact between free water (with dissolved oxygen) and zinc anodes. Chronoamperometry tests at a constant potential of − 150 mV were performed to investigate Zn^2+^ diffusion behaviors on anode surface. As shown in Fig. 3b, the current density of the bare Zn anodes continuously increases over 150 s, revealing a rampant 2D diffusion of Zn^2+^ after their adsorption on anode surface [22]. Due to such 2D diffusion behaviors, Zn^2+^ ions move to small protrusions (with high specific surface energy), accelerating the formation of large-sized dendrites, as illustrated in Fig. 3c. Differently, the Cu nanowire networks-protected Zn anodes display rapidly stabilized current density after the initial 10 s in their chronoamperometry curve (Fig. 3b). This reflects that lateral movement of Zn^2+^ on anode surface is constrained by the Cu nanowire networks. Since porous artificial interface layers on zinc anode surfaces have been widely proved to have the function of guiding Zn^2+^ flux and deposition sites [31, 32, 34], it is reasonable to believe that the Cu nanowire networks with porous nature homogenize Zn^2+^ ion concentration distribution and restrict rampant 2D diffusion of Zn^2+^ on anode surface (Fig. 3c). In fact, as will be discussed below, zincophilic Cu nanowires promote zinc nucleation/deposition and thus can reduce the number of Zn^2+^ ions accumulated at anode surface, also helping to restrict 2D diffusion of Zn^2+^. Besides, unstable electric field on the zinc anode surface during zinc plating/stripping processes is considered as another important factor in causing uneven Zn^2+^ ion concentration distribution and zinc dendrite growth [21], while for the Cu nanowire networks-protected Zn anodes, highly conductive Cu nanowires may favor the stabilization of surface electric field, thereby inhibiting zinc dendrite [34, 47].

The above analysis is verified by COMSOL Multiphysics finite element simulation (Fig. 3d-e). Since the surface of commercial Zn foil anodes cannot be ideally smooth, and meanwhile, zinc nucleus/clusters inevitably form during the zinc plating process, there are always some tiny protrusions on the zinc anode surface. Due to the “tip effect,” a strong localized electric field generates on the tip of these protrusions and then adsorbs Zn^2+^ through electrostatic forces to deposit on such sites. As the zinc plating process proceeds, these tiny protrusions grow into large dendrites (Fig. 3d). However, in the case of introducing the Cu nanowire networks on the zinc anode surface, nanoscaled Cu nanowires with large specific surface area are able to reduce local current density and homogenize Zn^2+^ concentration field (Fig. 3e). As a result, zinc dendrite growth is dramatically inhibited.

**Fig. 4:**
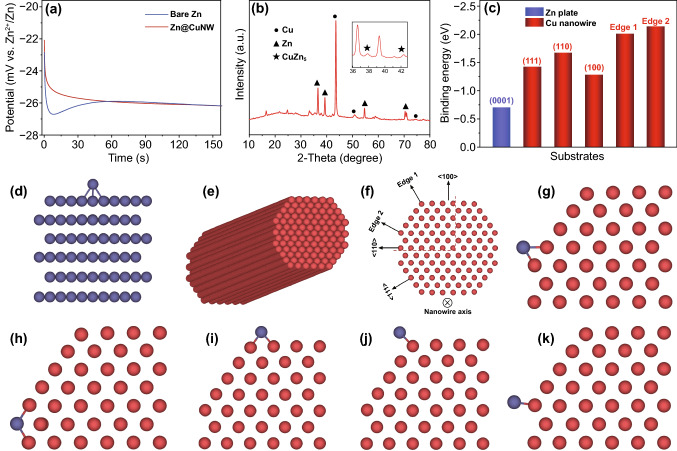
**a** Zinc deposition curves at 0.1 mA cm^−2^ on bare Zn anodes and Zn@CuNW anodes. **b** XRD pattern (inset: enlarged zone) of CuNW/membrane after depositing 1 mAh cm^−2^ zinc on the Zn@CuNW electrodes. **c** Binding energies between the zinc atom and different substrates of Zn plate and Cu nanowires. **d** Calculation model of zinc absorbed on (0001) facet of Zn plates. **e** Bird view and **f** atomic model of the single-crystalline Cu nanowire. Stable adsorption sites for one zinc atom on various facets and edges of the Cu nanowires: **g** (111) facet, **h** (110) facet, **(i)** (100) facet, **j** edge 1 and **k** edge 2. The Lyons blue and brownish red balls are Zn and Cu atoms, respectively

Furthermore, the zincophilic feature of Cu nanowires also plays an important role in optimizing zinc plating/stripping behaviors. As shown in Fig. 4a, electrodeposition of zinc on bare Zn anodes corresponds to a large nucleation overpotential, but the nucleation overpotential of zinc deposition on Zn@CuNW anodes is almost negligible. Meanwhile, after zinc deposition on Zn@CuNW anodes, metallic Zn and CuZn_5_ alloy (JCPDS No. 35–1151) are detected from the Cu nanowire networks (Figs. 4b and S12-S13). These reveal the tendency of zinc deposition on Cu nanowires [42].

In order to deeply understand zinc deposition behaviors on Cu nanowires, DFT calculations were conducted. As a baseline, we firstly calculated the binding energy between zinc atom and the (0001) surface of Zn plates, which is − 0.71 eV (Fig. 4c-d). To probe the interaction between zinc and Cu nanowires, we modeled Cu nanowires as a single crystal terminated by four (111) facets, two (110) facets and two (100) facets (Fig. 4e-f) [48, 49]. The most stable zinc adsorption sites on typical facets and edges of the Cu nanowires are summarized in Fig. 4g-k. Zinc deposition on the (111) facets of the Cu nanowires corresponds to the binding energy of − 1.30 eV, revealing that Cu nanowires are more zincophilic than Zn plates. Besides, the calculations point out that Cu–Zn solid solutions (CuZn_*x*_) tend to form during zinc deposition on high-index facets such as Cu(110), which strengthens the interaction between zinc and Cu nanowires to a higher level of − 1.69 eV. More importantly, for the Cu nanowires, their edge sites with un-fully coordinated atoms are found to be highly active for zinc deposition, with binding energy values of − 2.01 eV for the edge < 111,100 > (i.e., the intersection of (111) and (100) facets) and − 2.14 eV for the edge < 111,110 > (i.e., the intersection of (111) and (110) facets). Therefore, although zinc deposition on the flat facets of Cu nanowires and on the flat facets of Cu plates exhibits similar binding energy values (Fig. S14), Cu nanowires with abundant edge sites are more advantageous than Cu plates as zincophilic materials to induce the uniform nucleation/deposition of zinc. This well explains that the Cu nanowire networks-protected zinc anodes exhibit much better electrochemical performance than previously reported Cu plate-supported zinc anodes (Table S1). Moreover, the easy nucleation/deposition of zinc on Cu nanowires also contributes to the constrained 2D diffusion of Zn^2+^ on Zn@CuNW anodes, since it helps to reduce the number of Zn^2+^ ions accumulated at anode/electrolyte interface.

**Fig. 5:**
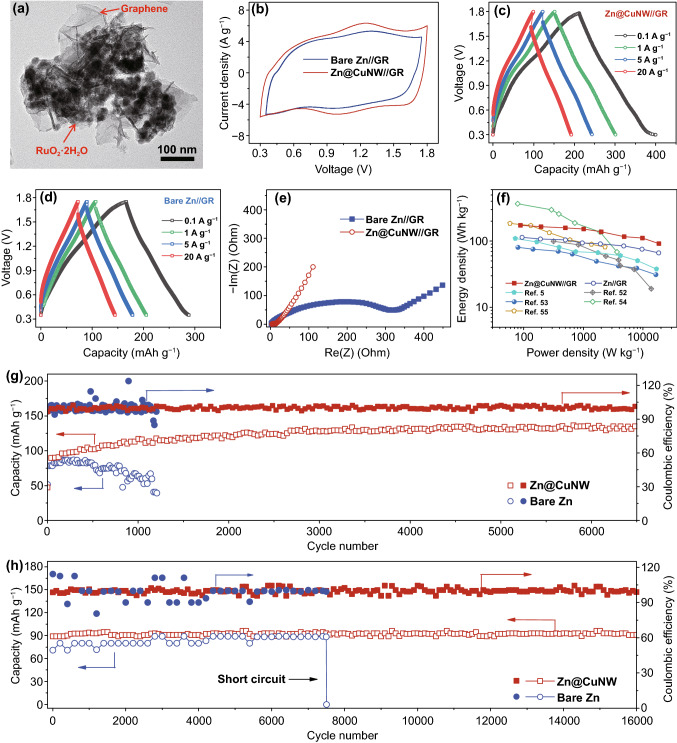
**a** TEM image of the GR nanocomposite. Electrochemical properties of the Zn@CuNW//GR and the bare Zn//GR zinc-ion hybrid supercapacitors: **b** CV curves at 20 mV s^−1^. **c**, **d** GCD curves at different current densities. **e** EIS spectra. **f** Ragone plots and long-term cycling behaviors at **g** 5 and **h** 20 A g^−1^

The long-term stable and high-rate Zn@CuNW anodes enable high-performance Zn-based EES devices. As a demonstration, we assembled zinc-ion hybrid supercapacitors with the Zn@CuNW anode, GR and 2 M ZnSO_4_ aqueous electrolyte (Fig. 5). Physicochemical characteristics of the GR nanocomposite are provided in Figs. 5a and S15-S18. In the GR nanocomposite, amorphous RuO_2_·2H_2_O nanoparticles are loaded on graphene nanosheets. It needs to emphasize that the existence of structural water is essential for ruthenium oxides to store Zn^2+^ ions through a pseudocapacitive mechanism [50]. For the assembled Zn@CuNW//GR zinc-ion hybrid supercapacitors, they can be charged/discharged in the voltage window of 0.3–1.8 V (Figs. 5b and S19), corresponding to pseudocapacitive storage of Zn^2+^ by RuO_2_·2H_2_O and ion adsorption/desorption on graphene surface [50, 51]. A high discharge capacity of 197 mAh g^−1^ is achieved (Fig. 5c). In addition, the Zn@CuNW//GR zinc-ion hybrid supercapacitors exhibit impressive rate capability, with 99 mAh g^−1^ discharge capacity at a current density of 20 A g^−1^ (i.e., a charge/discharge cycle only needs 36 s). This is because electrochemical energy storage of the Zn@CuNW//GR zinc-ion hybrid supercapacitors is dominated by fast capacitive behaviors, instead of relatively slow diffusion-controlled behaviors, as discussed in Fig. S20. Electrochemical impedance spectroscopy (EIS) spectra reveal the very small charge transfer resistance during energy storage processes of the Zn@CuNW//GR zinc-ion hybrid supercapacitors (Fig. 5e), which well explains the outstanding rate performance. As a result, maximum energy density and power density of the Zn@CuNW//GR zinc-ion hybrid supercapacitors are as high as 174 Wh kg^−1^ and 18 kW kg^−1^, respectively (Fig. 5f), suggesting their better rate capability than zinc-ion batteries and superior charge storage ability than carbon based zinc-ion hybrid supercapacitors [24, 52–54]. Moreover, after 6,500 charge/discharge cycles at 5 A g^−1^ and 16,000 cycles at 20 A g^−1^, the Zn@CuNW//GR zinc-ion hybrid supercapacitors do not show obvious decay in capacity, and corresponding coulombic efficiency maintains ~ 100% during the long-term cycling tests, indicating superior cyclic stability and high reversibility (Fig. 5g-h). The slight increase in capacity in the initial 1500 cycles in Fig. 5g is caused by electrode activation. That is, electrolytes gradually infiltrate into the whole electrode and more electrochemically active materials can participate in electrochemical reactions during repeated charge/discharge processes [55, 56].

In sharp contrast, when Zn@CuNW anodes are replaced by bare Zn anodes, assembled bare Zn//GR zinc-ion hybrid supercapacitors display inferior electrochemical properties, such as narrower working voltage window of 0.35–1.75 V (note that gas evolution occurs if a wider working voltage window such as 0.3–1.8 V is applied, which is associated with unstable zinc plating behavior on bare zinc anodes), smaller discharge capacity, notably increased electrochemical impedance and seriously deteriorated cycling performance (Figs. 5b-h and S21-S22). These confirm the superior long-term stability and good rate performance of the Zn@CuNW anodes in Zn-based EES systems, thereby emphasizing again the validity of using Cu nanowire networks to protect zinc anodes.

## Conclusions

In summary, zincophilic Cu nanowire networks were employed to stabilize zinc anodes from multiple aspects. For the Cu nanowire networks covering on the zinc anode surface, their porous nature guided Zn^2+^ flux, and meanwhile, their large specific surface area endowed by nanoscale size helps to reduce local current density and homogenize Zn^2+^ concentration field. Besides, the hydrophobic feature of the Cu nanowire networks suppressed the direct contact between free water (with dissolved oxygen) and zinc anodes, inhibiting side reactions such as anode corrosion and hydrogen evolution. It was revealed that the facets and edge sites of the Cu nanowires, especially the latter ones, were highly zincophilic to induce uniform zinc nucleation/deposition. The above all aspects work together to alleviate zinc dendrite and side reactions. As a result, the Cu nanowire networks-protected zinc anodes exhibited an ultralong cycle life of over 2,800 h and could continuously operate for hundreds of hours even at large charge/discharge currents and areal capacities, remarkably superior to bare zinc anodes and most of currently reported zinc anodes. Moreover, zinc-ion hybrid capacitors were constructed with the Cu nanowire networks-protected zinc anodes, and high capacity, over 16,000-cycle lifespan and rapid charge/discharge ability were achieved. This work provides new thoughts to realize high-performance zinc anodes for Zn-based EES.

## Supplementary Information

Below is the link to the electronic supplementary material.

Supplementary file1 (PDF 2045 KB)
