# Supplementary material for: Stable Zinc Anodes Enabled by Zincophilic Cu Nanowire Networks
Source: Nanomicro Lett. 2021 Dec 23;14:39. doi: 10.1007/s40820-021-00783-4 (PMC8702588; doi:10.1007/s40820-021-00783-4)
Supplement: Supplementary file 1 — Supplementary file1 (PDF 2045 KB) [file 40820_2021_783_MOESM1_ESM.pdf]

Supporting Information for

Stable Zinc Anodes Enabled by Zincophilic Cu Nanowire Networks

Shiyin Xie[1, #], Yang Li[1, #], Xu Li[1], Yujun Zhou[1], Ziqi Dang[1], Jianhua Rong[1], and Liubing Dong[1, *]

[1]College of Chemistry and Materials Science, Jinan University, Guangzhou 511443, P. R. China

[#]Shiyin Xie and Yang Li contributed equally to this work.

*Corresponding author. E-mail: donglb@jnu.edu.cn (L. Dong)

S1 Experimental Section

S1.1 COMSOL Simulation

We applied the present nonlinear phase-field model to a realistic electrodeposition system, *i.e.*, zinc plating on Zn foil anodes [S1]. The Zn^{2+} concentration distribution and diffusion behavior were investigated using COMSOL multi-physics finite element method (FEM). Two geometric models were established to simulate the bare Zn foil and the Zn@CuNW structure. The migration of Zn^{2+} driven by electric field and diffusion flow in both electrolytes and solid phase are considered during these simplified simulations. Therefore, two physical models of electrostatic and transport of diluted species based on the partial differential equations listed below were coupled to conduct FEM simulation [S2, S3].

$$E = -\nabla \phi \tag{S1}$$

$$N = -D\nabla = c + ucE \tag{S2}$$

$$\frac{\partial c}{\partial t} = -\nabla N \tag{S3}$$

where ϕ is electric potential, E is electric field, D is Zn^{2+} diffusion coefficient, c is Zn^{2+} concentration, u is Zn^{2+} ionic mobility in electrolytes, and N is Zn^{2+} flux vector and t is diffusion time. The potential difference ($\Delta\phi$) through these electrolytes was set as 10 mV. To investigate Zn^{2+} ion transport behaviors with limited liquid electrolytes in long time cycling, the same physical model was established and the ratio of diffusion coefficients of Zn^{2+} in liquid electrolytes and solid particles was decreased to 10.0. The mobility of Zn^{2+} (m) for liquid electrolyte and solid particles was defined by the Nernst-Einstein equation. The bottom boundaries of two simulation areas were the Dirichlet boundaries with ϕ_0 of 0 V and c_0 of 0 M. The top boundaries of two simulation areas were also Dirichlet boundaries with ϕ_1 of 10 mV and c_1 of 1.0 M. The other boundaries were natural boundaries with zero flux. Herein, the phase-field model was implemented through FEM simulations based on the COMSOL Multiphysics.

S1.2 DFT Calculations

Spin-polarized DFT calculations were employed to investigate the adsorption of single zinc atom on different substrates. The calculations were performed using the Vienna *ab initio* Simulation Package [S4-S6], using the Perdew, Burke, and Ernzerhof (PBE) version of the generalized gradient approximation (GGA) as the exchange-correlation functional [S7], with the projector augmented wave (PAW) approximation representing the atomic core regions [S8]. The simulation was taken using the substrate surface with a seven-layer supercell and relaxing the top three-layer atoms. Adsorption of one zinc atom corresponded to a coverage of 0.0625

ML on the surface. An atomic force convergence criterion of 0.01 eV Å^{-1} was used to identify optimized geometries. Plane wave basis sets were truncated at a kinetic energy cutoff of 450 eV, and a vacuum layer of 15 Å was chosen in the *z* direction to eliminate the interaction between its periodic replicas. The Brillouin zone was sampled on the basis of the Monkhorst-Pack scheme with a 3×3×1 *k*-point mesh [S9]. In addition, to account for the Coulombic repulsion between localized electrons in Cu and Zn, the DFT+U schema was used.[S10] The Hubbard parameters (U-J) for Cu and Zn are set as 5 and 7.5 eV, respectively [S11]. The initial position of the zinc atom was placed at the apex of the polyhedral convex cone by the closest packing to identify the most favorable adsorption geometry for each metallic surface model and at the bridge sites on the Cu nanowire edge. A dipole correction was used to correct the unphysical interaction between dipoles in adjacent images along the surface normal for asymmetric slab models [S12]. The binding energy (ΔE_b) was calculated by the Eq. (S4),

$$\Delta E_b = E_{sub+Zn} - E_{sub} - E_{Zn} \quad \text{(S4)}$$

where E_{sub+Zn} represents the total energy of metal substrate combined with one zinc atom, E_{sub} and E_{Zn} are the energy of the substrate and the energy of one zinc atom, respectively.

S2 Supplementary Figures and Table

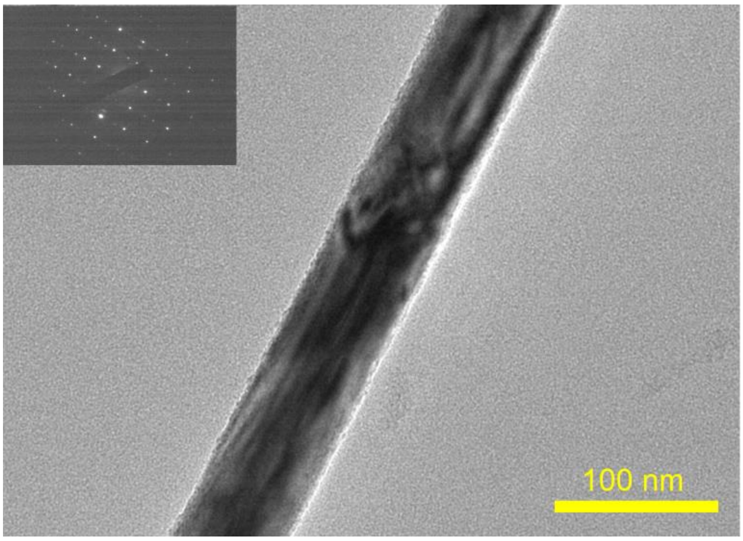

Fig. S1 TEM image and selected-area electron diffraction (SAED) pattern (inset) of the synthesized Cu nanowires

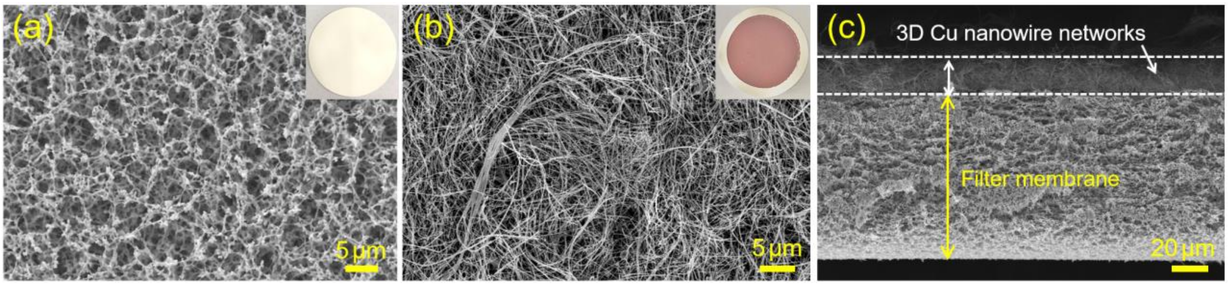

Fig. S2 (**a**) SEM image of pristine filter membrane. (**b**) Surface and (**c**) cross-sectional SEM images of the fabricated CuNW/membranes with loading of 0.4 mg cm^{-2}. Insets in (**a**) and (**b**) are digital photos of pristine filter membrane and the CuNW/membranes, respectively

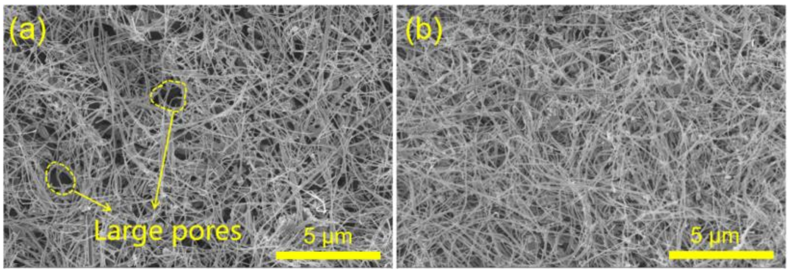

Fig. S3 SEM images of the CuNW/membranes, in which mass loading of Cu nanowires is (**a**) 0.2 and (**b**) 1.2 mg cm^{-2}. When the mass loading is too low (*e.g.*, 0.2 mg cm^{-2}), the Cu nanowires do not form a uniform and continuous network (there are some large holes in the Cu nanowire network). Therefore, the Cu nanowire network is difficult to homogenize surface electric/ionic field and Zn^{2+} concentration field, and thus cannot effectively stabilize Zn anodes. When the mass loading is high (*e.g.*, 1.2 mg cm^{-2}), the Cu nanowires are able to form a continuous network . However, relatively large thickness (~27 μm) and dense arrangement of the hydrophobic Cu nanowire network increase transport resistance of Zn^{2+}, thus leading to increased voltage hysteresis and modest cycling stability of corresponding Zn anodes

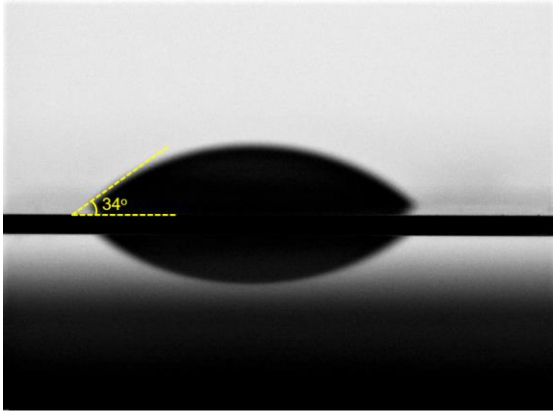

Fig. S4 Water contact angle of pristine filter membranes

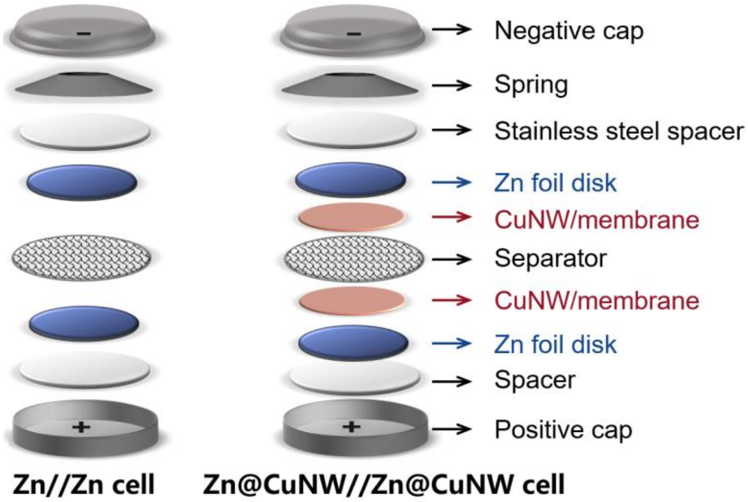

Fig. S5 Schematic diagrams of symmetric cells with bare Zn foil electrodes and Cu nanowire networks-protected Zn foil electrodes. In the symmetric cells with Cu nanowire networks-protected Zn foil electrodes, Cu nanowire networks directly contacted with Zn foils (*i.e.*, the filter membrane substrates of the CuNW/membranes contacted with the separator). The used electrolyte was 2 M $ZnSO_4$ aqueous solution. We also replaced the CuNW/membranes with pristine filter membranes to assemble symmetric cells (Fig. S4)

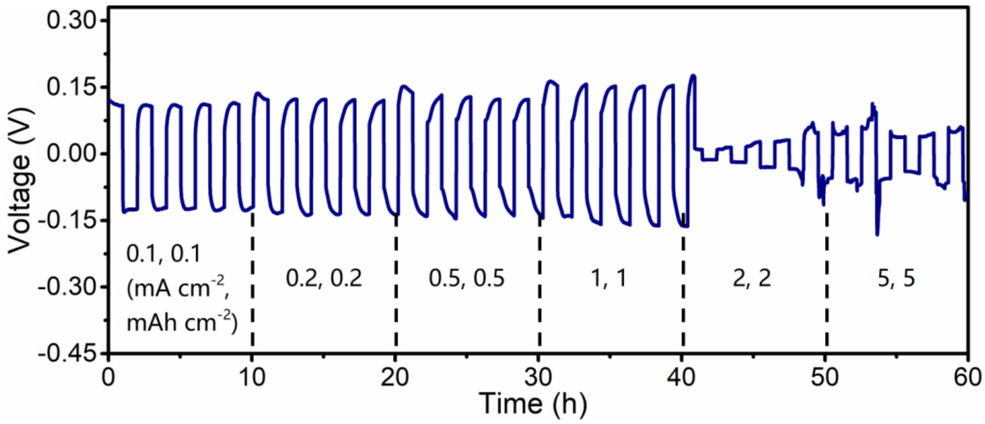

Fig. S6 Rate performance of the symmetric cells with pristine filter membrane-protected Zn foil electrodes (*i.e.*, replacing the CuNW/membranes in Zn@CuNW//Zn@CuNW symmetric cells with pristine filter membranes). The symmetric cells show large voltage hysteresis and cannot operate at large currents and areal capacities. This suggests that the Cu nanowire networks, instead of the filter membrane substrate, play critical role in CuNW/membrane protecting zinc anodes

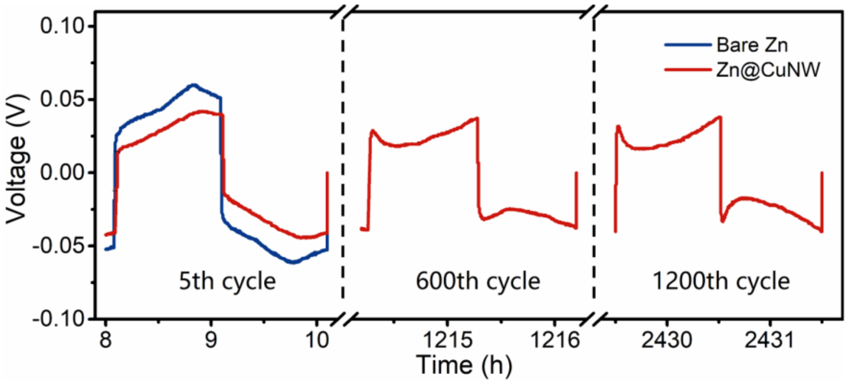

Fig. S7 The 5th, 600th and 1200th cycle GCD profiles during long-term cycling stability of the symmetric cells at 0.2 mA cm^{-2} and 0.2 mAh cm^{-2}

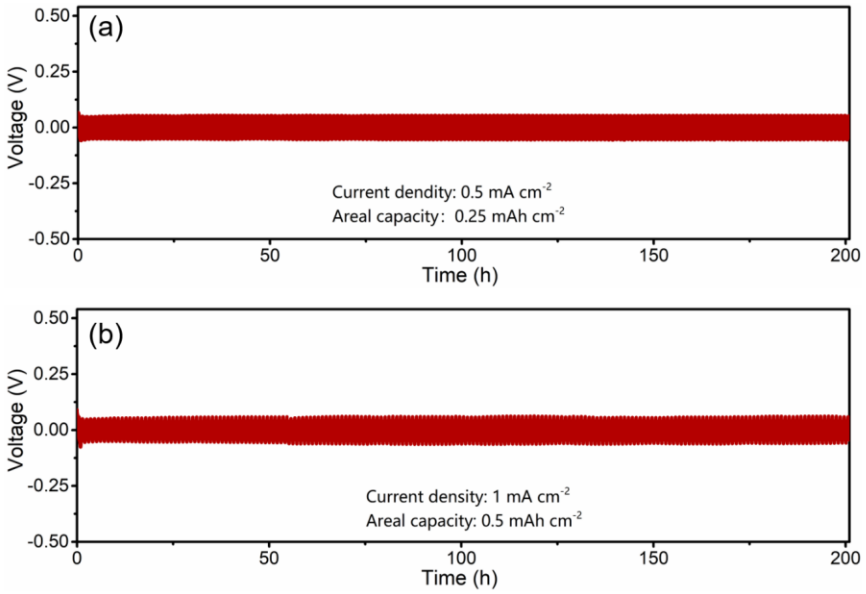

Fig. S8 Cycling stability of Zn@CuNW//Zn@CuNW symmetric cells at different current densities and areal capacities: (**a**) 0.5 mA cm^{-2} and 0.25 mAh cm^{-2}; (**b**) 1 mA cm^{-2} and 0.5 mAh cm^{-2}

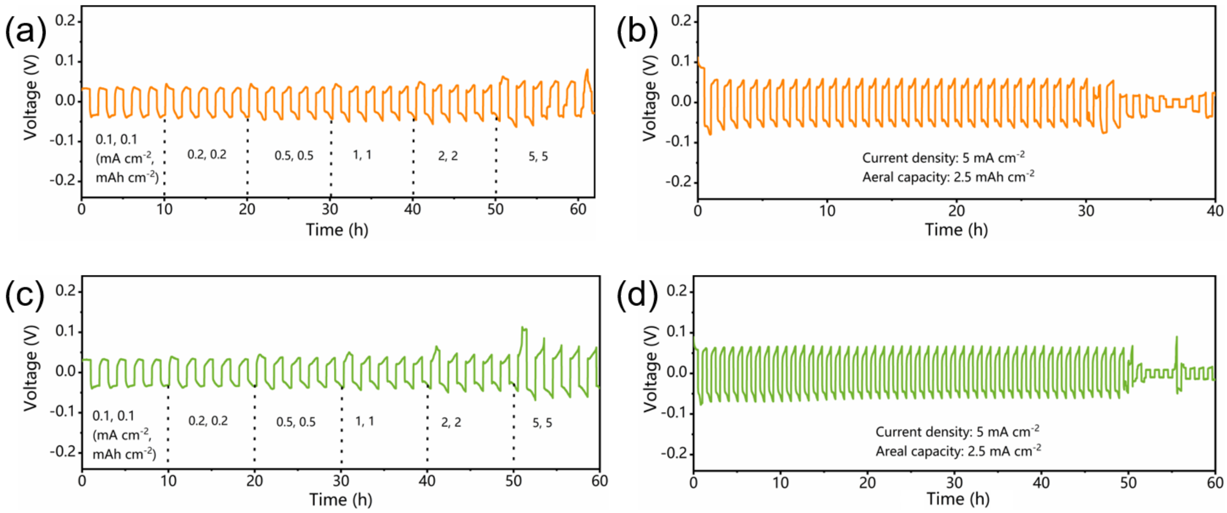

Fig. S9 Rate performance and cycling stability of symmetric cells with Cu nanowire networks-stabilized zinc electrodes, in which mass loading of the Cu nanowires is (**a-b**) 0.2 and (**c-d**) 1.2 mg cm^{-2}

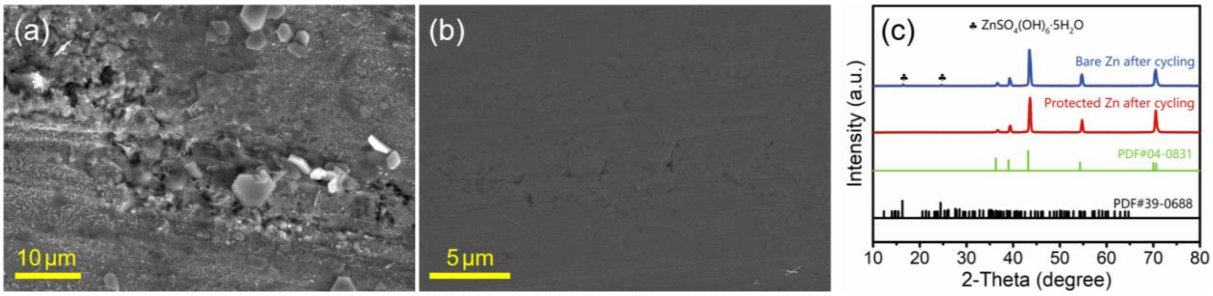

Fig. S10 SEM images of (**a**) bare Zn foil after cycling for 50 hours and (**b**) protected Zn foil after cycling for 50 hours. (**c**) Corresponding XRD patterns. These Zn foil electrodes were repeatedly charged/discharged at a current density of 5 mA cm^{-2} and areal capacity of 2.5 mAh cm^{-2}. After cycling, large dendrites/protuberances appear on the surface of the bare Zn foil electrode, accompanying with the formation of $ZnSO_4(OH)_6 \cdot 5H_2O$ by-products (JCPDS#39-0688). Differently, the protected Zn foil electrode after cycling shows a relatively flat surface and no by-products are detected by XRD analysis

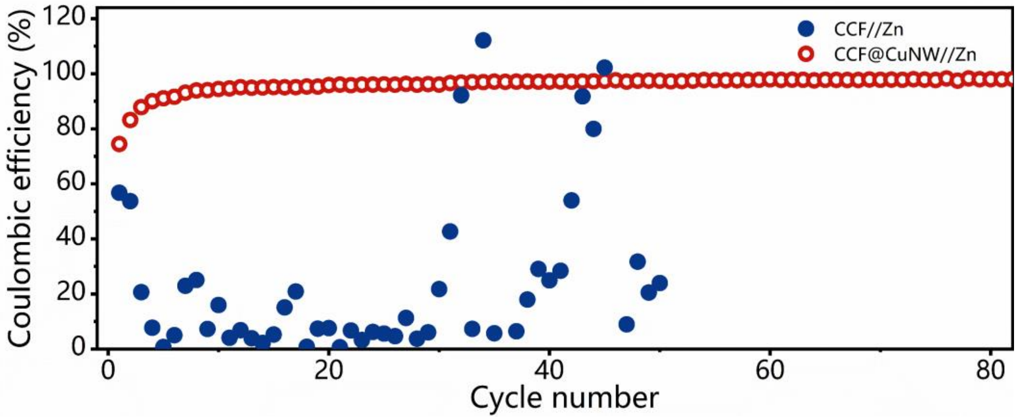

Fig. S11 Zinc deposition/stripping Coulombic efficiency (CE) at current density of 1 mA cm^{-2} and areal capacity of 0.5 mAh cm^{-2}. We measured the CE values by assembling cells with the negative electrode of Zn foil and positive electrode of carbon-coated Cu foil (CCF) or Cu nanowire networks-stabilized CCF foil (denoted as CCF@CuNW). The CCF@CuNW//Zn cells exhibit much higher CE values than the CCF//Zn cells, indicating that the Cu nanowire networks significantly improve zinc deposition/stripping efficiency

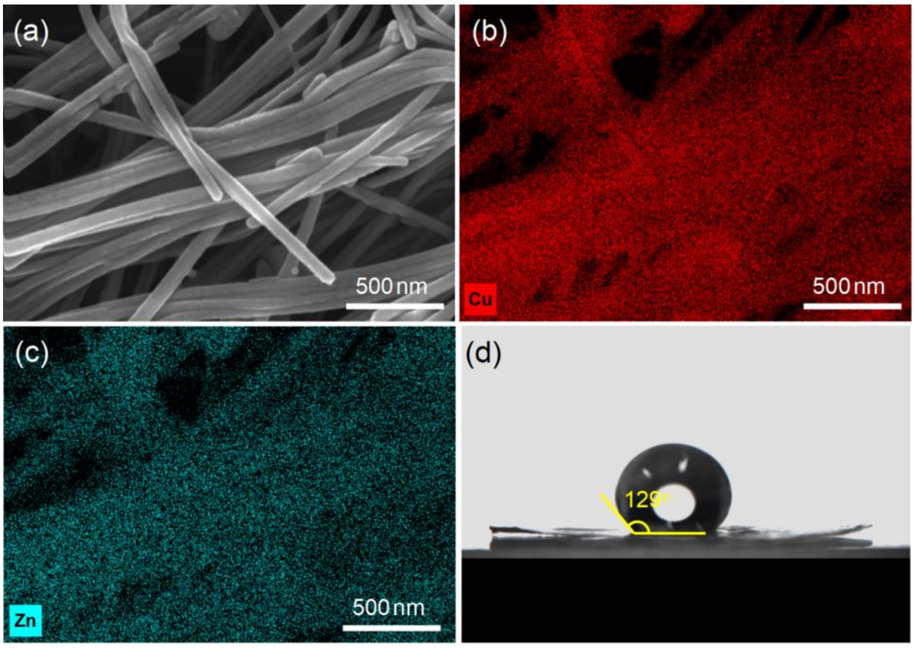

Fig. S12 (**a**) SEM image, EDS mapping of (**b**) Cu and (**c**) Zn elements and (**d**) water contact angle of the Cu nanowire networks after depositing 1 mAh cm^{-2} Zn on the Zn@CuNW anodes. Compared with pristine Cu nanowires in Fig. 1b (in the main text), the Cu nanowires in (**a**) exhibit increased diameter, and meanwhile, Zn element is detected, confirming the uniform nucleation/deposition of zinc on the surface of Cu nanowires, which greatly helps to suppress zinc dendrite growth. Meanwhile, the Cu nanowire networks keep their 3D porous structure and hydrophobic feature after depositing 1 mAh cm^{-2} zinc on the Zn@CuNW electrodes, in which case the Cu nanowire networks still possess the ability to stabilize zinc anode through reducing local current density, homogenizing surface electric field and inhibiting side reactions.

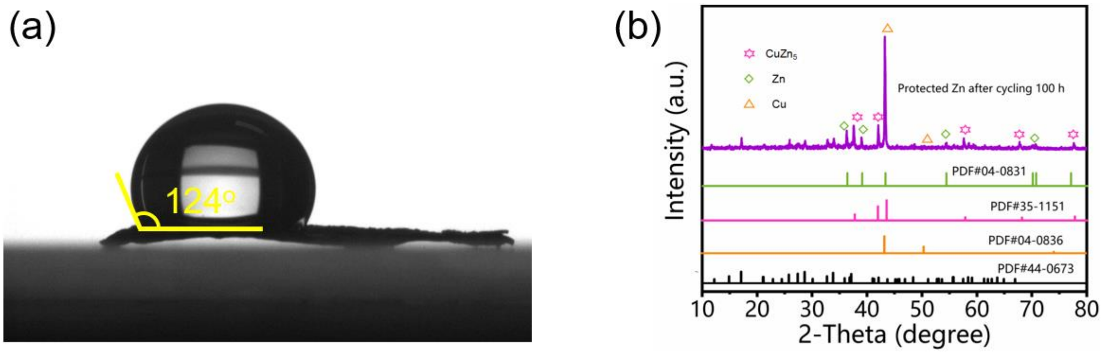

Fig. S13 (**a**) Water contact angle and (**b**) XRD pattern of the CuNW/membranes after cycling 100 h (test conditions: 5 mA cm^{-2} and 2.5 mAh cm^{-2}) in Zn@CuNW//Zn@CuNW symmetric cells. These unlabeled diffraction peaks in the XRD pattern derive from $Zn_4SO_4(OH)_6 \cdot 4H_2O$ (PDF#44-0673). Water contact angle of the CuNW/membranes is 124°, suggesting that the CuNW/membranes are still hydrophobic (decreased water contact angle of the CuNW/membranes than their pristine state is associated with the existence of metallic zinc and $CuZn_5$ alloy on the surface of Cu nanowires after charge/discharge cycling). Meanwhile, XRD pattern of the CuNW/membranes does not present characteristic diffraction peaks of copper oxides and copper hydroxides. Therefore, it is reasonable to believe that in our Zn-based energy storage systems, oxidization of the Cu nanowires is a very slow process, and protective effects of the Cu nanowire networks on zinc anodes can maintain for a long period

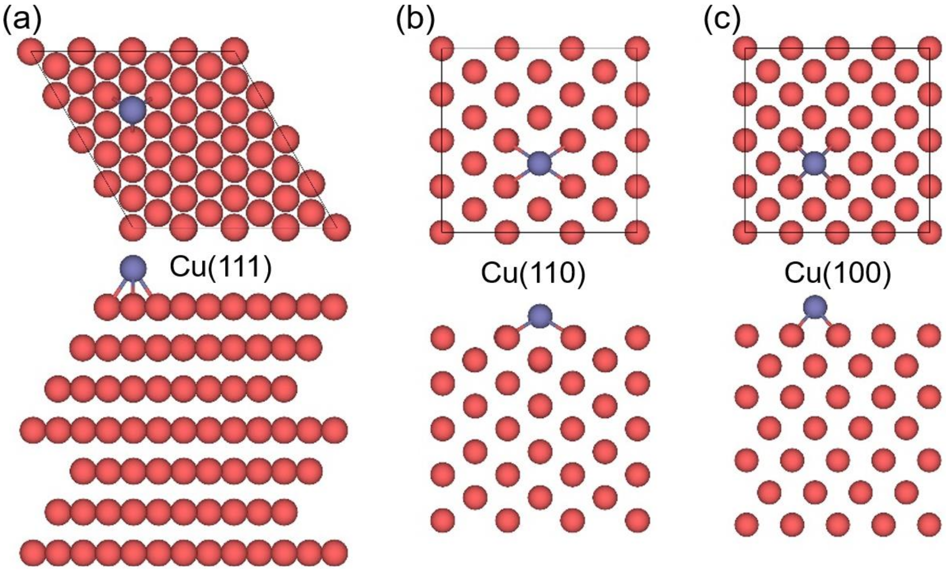

Fig. S14 Calculation models of zinc absorbed on various facets of Cu plates: (**a**) (111), (**b**) (110) and (**c**) (100). The lyons blue balls are Zn atoms and the brownish red balls are Cu atoms. Binding energy of zinc atom adsorbed on the (111), (110) and (100) facets is calculated to be -1.43, -1.68 and -1.29 eV, respectively

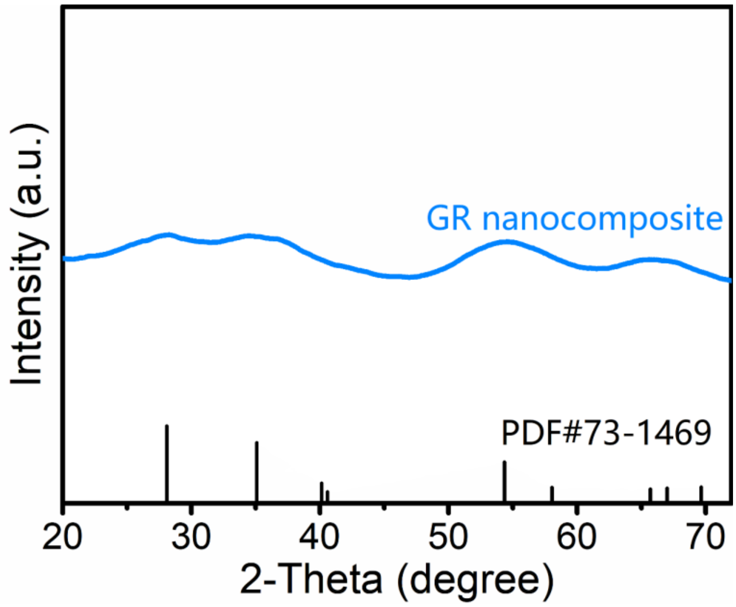

Fig. S15. XRD pattern of the synthesized GR nanocomposite. The broad diffraction peaks at 28°, 35°, 54° and so on reveal the presence of ruthenium oxides (JCPDS#73-1469) with low crystallinity. Diffraction peaks of graphene are not obvious in the XRD pattern, which is likely to be caused by its low content in the GR nanocomposite and relatively weak characteristic peaks for graphene materials. However, the existence of graphene is confirmed through TEM observation in Fig. 5a and other methods displayed in Fig. S16-S18

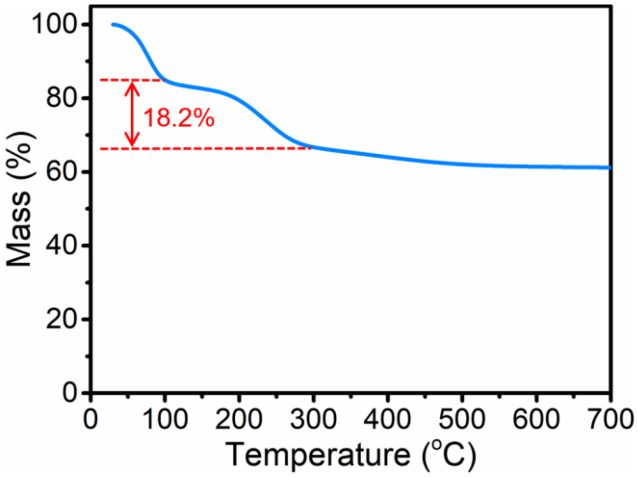

Fig. S16 TG curve of the GR nanocomposite. The mass loss in the temperature range of 30-100, 100-300 and 300-700 °C corresponds to the removal of free water, structural water and carbon (*i.e.*, graphene), respectively. It can be calculated that each $RuO_2 \cdot xH_2O$ molecule contains 2 water molecules (*i.e.*, x=2), and graphene content in the GR nanocomposite (excluding free water) is 6.6 wt%

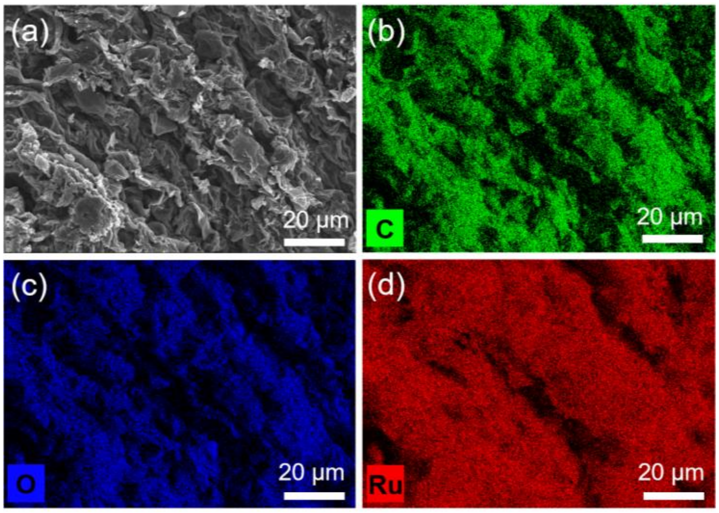

Fig. S17 (**a**) SEM image and EDS mapping of (**b**) C, (**c**) O and (**d**) Ru elements of the GR nanocomposite

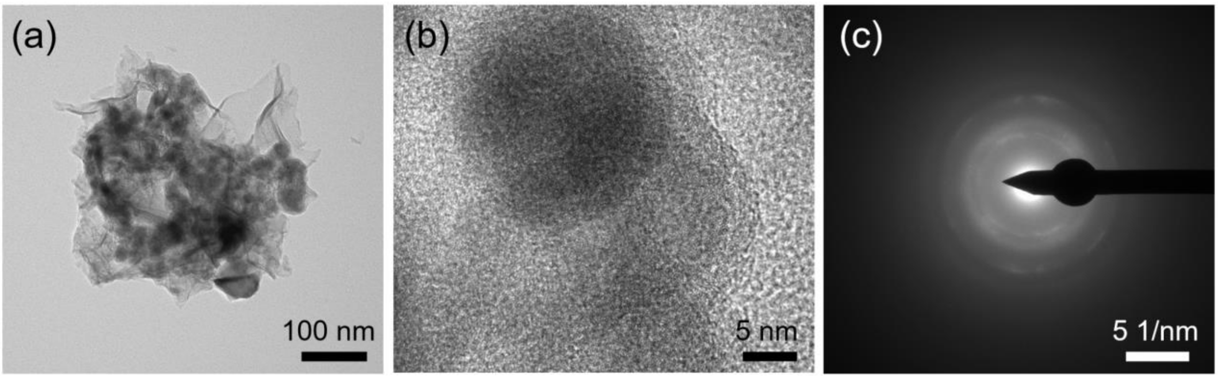

Fig. S18 (**a**) TEM image, (**b**) high-resolution TEM image and (**c**) SAED image of the GR nanocomposite. The absence of continuous lattice fringes in the high-resolution TEM image and the halo ring pattern in the SAED image demonstrate the amorphous feature of the $RuO_2 \cdot xH_2O$ component in the GR nanocomposite

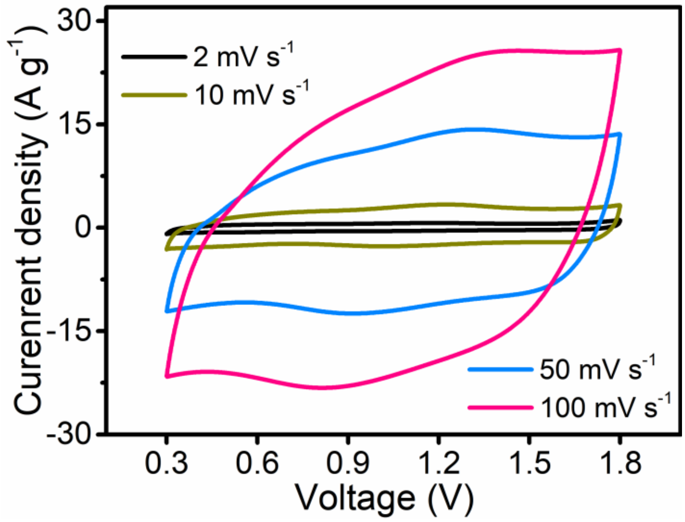

Fig. S19 CV curves of the Zn@CuNW//GR zinc-ion hybrid supercapacitors

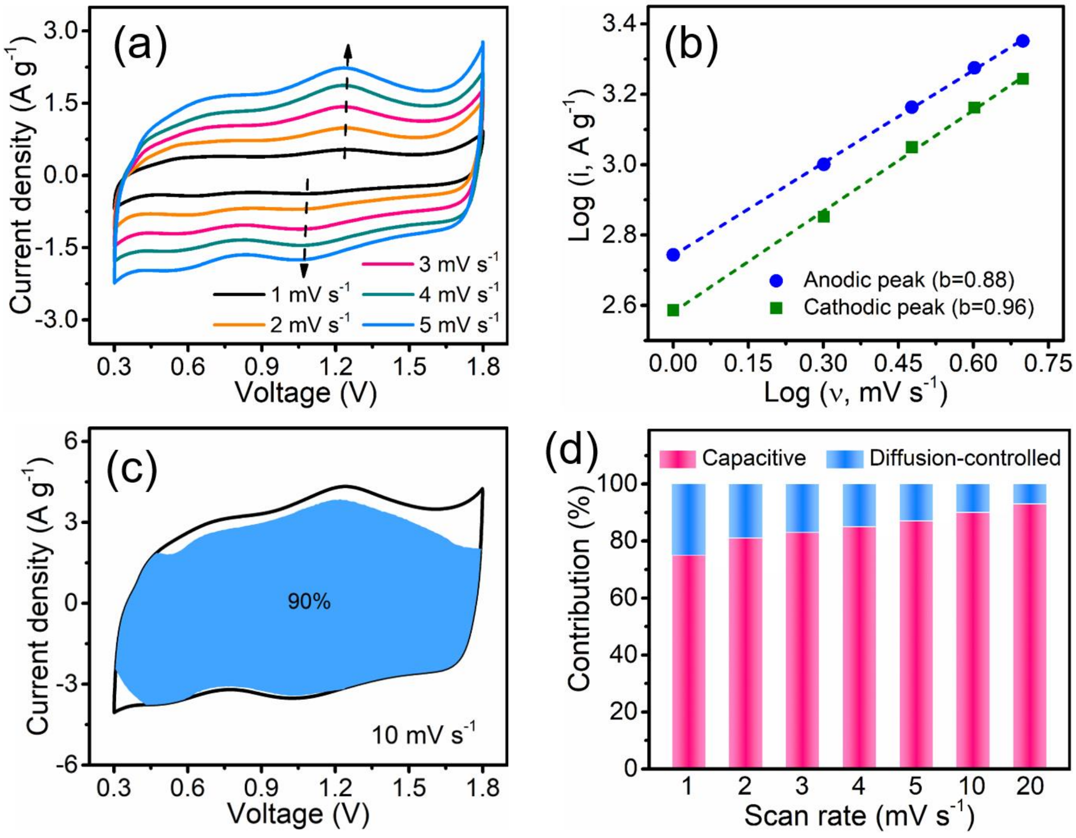

Fig. S20 Electrochemical kinetic analysis of the Zn@CuNW//GR zinc-ion hybrid supercapacitors: (**a**) CV curves at low scan rates; (**b**) relationship analysis between scan rate and peak current; (**c**) CV curve at 10 mV s^{-1} and the current contributed by capacitive process (blue area); (**d**) capacity contribution of capacitive process and diffusion-controlled process at various scan rates. By analyzing the relationship between scan rate and peak current, *b* values of anodic peaks and cathodic peaks are determined to be 0.88 and 0.96, respectively.[S13,S14] This means that energy storage of the Zn@CuNW//GR zinc-ion hybrid supercapacitors is dominated by capacitive behaviors. Quantitative analysis in (**c**) and (**d**) also reveals that capacity contribution of capacitive process is much higher than that of diffusion-controlled process. Therefore, the Zn@CuNW//GR zinc-ion hybrid supercapacitors are endowed with outstanding rate capability.

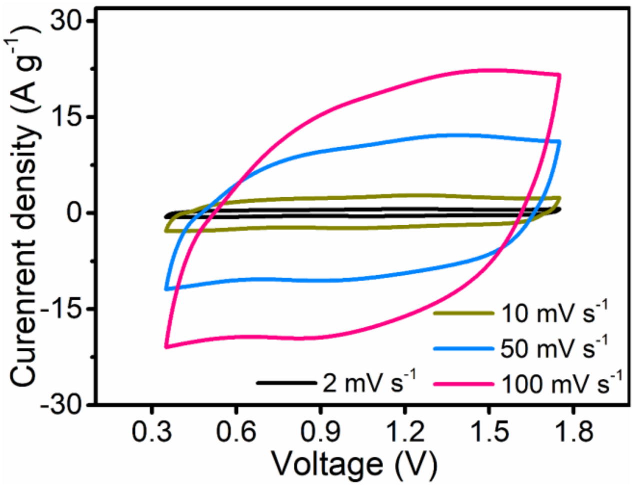

Fig. S21 CV curves of the bare Zn//GR zinc-ion hybrid supercapacitors. The hybrid supercapacitors possess a working voltage window of 0.35-1.75 V

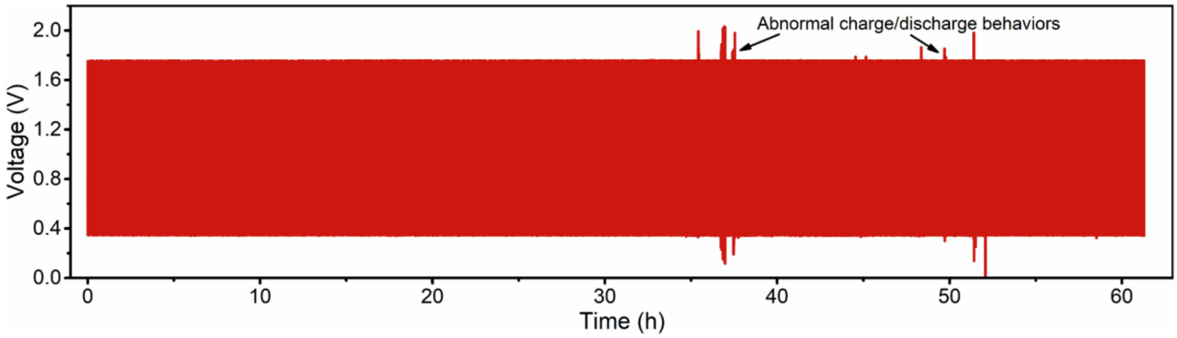

Fig. S22 GCD curves during cycling tests at 5 A g^{-1} of the bare Zn//GR zinc-ion hybrid supercapacitors. After repeated charge/discharge cycles for ~35 hours, the GCD profiles become abnormal, which is ascribed to the failure of bare Zn anodes (because for the Zn@CuNW//GR zinc-ion hybrid supercapacitors, they can be repeatedly charged/discharged for a much longer time, proving that the GR cathode is electrochemically stable enough)

Table S1 Comparison of electrochemical properties of previously reported zinc anodes

**Zinc anodes**	**Test conditions**			**Lifespan**	***Refs.***
	**Current density**	**Areal capacity**	**Cumulative plated capacity**		
3D copper nanowire networks-protected zinc foil	0.2 mA cm^{-2}	0.2 mAh cm^{-2}	560 mAh cm^{-2}	2800 h	This work
	5 mA cm^{-2}	2.5 mAh cm^{-2}	140 mAh cm^{-2}	280 h	
	10 mA cm^{-2}	5 mAh cm^{-2}	65 mAh cm^{-2}	130 h	
Copper foam-supported zinc anodes	2 mA cm^{-2}	1 mAh cm^{-2}	75 mAh cm^{-2}	150 h	[S15]
Porous copper-supported zinc anodes	0.5 mA cm^{-2}	0.5 mAh cm^{-2}	350 mAh cm^{-2}	350 h	[S16]

$Ti_3C_2T_x$ MXene@Zn paper	1 mA cm^{-2}	1 mAh cm^{-2}	300 mAh cm^{-2}	300 h	[S17]
CNT/carbon fiber supported zinc anodes	2 mA cm^{-2}	2 mAh cm^{-2}	200 mAh cm^{-2}	200 h	[S18]
	5 mA cm^{-2}	2.5 mAh cm^{-2}	87.5 mAh cm^{-2}	175 h	
N-doped carbon-coated zinc foil	2 mA cm^{-2}	2 mAh cm^{-2}	400 mAh cm^{-2}	400 h	[S19]
In-coated zinc foil	1 mA cm^{-2}	1 mAh cm^{-2}	300 mAh cm^{-2}	300 h	[S20]
	5 mA cm^{-2}	1 mAh cm^{-2}	500 mAh cm^{-2}	100 h	
rGO-coated zinc foil	1 mA cm^{-2}	1 mAh cm^{-2}	1200 mAh cm^{-2}	1200 h	[S21]
Ultrathin TiO_2-coated zinc foil	1 mA cm^{-2}	1 mAh cm^{-2}	150 mAh cm^{-2}	150 h	[S22]
Al_2O_3-coated zinc foil	1 mA cm^{-2}	1 mAh cm^{-2}	500 mAh cm^{-2}	500 h	[S23]
Zn@carbon fiber	1 mA cm^{-2}	1 mAh cm^{-2}	175 mAh cm^{-2}	175 h	[S24]
Carbon black-coated zinc foil	0.5 mA cm^{-2}	0.5 mAh cm^{-2}	400 mAh cm^{-2}	400 h	[S25]
ZnF_2 coated-zinc foil	10 mA cm^{-2}	10 mAh cm^{-2}	590 mAh cm^{-2}	590 h	[S26]

Supplementary References

[S1] L. Chen, H.W. Zhang, L.Y. Liang, Z. Liu, Y. Qi et al., Modulation of dendritic patterns during electrodeposition: a nonlinear phase-field model. J. Power Sources **300**, 376-385 (2015). https://doi.org/10.1016/j.jpowsour.2015.09.055

[S2] J. Newman, K.E. Thomas, H. Hafezi, D.R. Wheeler, Modeling of lithium-ion batteries. J. Power Sources **119**, 838-843 (2003). https://doi.org/10.1016/S0378-7753(03)00282-9

[S3] J. Newman, K.E. Thomas-Alyea, *Electrochemical Systems* (3rd Edition). Wiley, Hoboken, (2004).

[S4] G. Kresse, J. Hafner, Ab initio molecular dynamics for open-shell transition metals. Phys. Rev. B **48**, 13115 (1993). https://doi.org/10.1103/PhysRevB.48.13115

[S5] G. Kresse, J. Furthmüller, Efficiency of ab-initio total energy calculations for metals and semiconductors using a plane-wave basis set. Comput. Mater. Sci. **6**(1), 15-50 (1996). https://doi.org/10.1016/0927-0256(96)00008-0

[S6] G. Kresse, J. Furthmüller, Efficient iterative schemes for ab initio total-energy calculations using a plane-wave basis set. Phys. Rev. B **54**, 11169 (1996). https://doi.org/10.1103/PhysRevB.54.11169

[S7] J.P. Perdew, K. Burke, M. Ernzerhof, Generalized gradient approximation made simple. Phys. Rev. Lett. **77**, 3865 (1996). https://doi.org/10.1103/PhysRevLett.77.3865

[S8] P.E. Blöchl, Projector augmented-wave method. Physi. Rev. B **50**, 17953 (1994). https://doi.org/10.1103/PhysRevB.50.17953

[S9] H.J. Monkhorst, J.D. Pack, Special points for brillouin-zone integrations. Phys. Rev. B **13**, 5188 (1976). https://doi.org/10.1103/PhysRevB.13.5188

[S10] S.L. Dudarev, G.A. Botton, S.Y. Savrasov, C.J. Humphreys, A.P. Sutton, Electron-energy-loss spectra and the structural stability of nickel oxide: an LSDA+U study. Phys. Rev. B **157**, 1505 (1998). https://doi.org/10.1103/PhysRevB.57.1505

[S11] T. Foroozan, V. Yurkiv, S. Sharifi-Asl, R. Rojaee, F. Mashayek et al., Non-dendritic Zn electrodeposition enabled by zincophilic graphene substrates. ACS Appl. Mater. Interfaces **11**(47), 44077-44089 (2019). https://doi.org/10.1021/acsami.9b13174

[S12] J. Neugebauer, M. Scheffler, Adsorbate-substrate and adsorbate-adsorbate interactions of Na and K adlayers on Al(111). Phys. Rev. B **46**, 16067 (1992). https://doi.org/10.1103/PhysRevB.46.16067

[S13] L. Dong, W. Yang, W. Yang, C. Wang, Y. Li et al., High-power and ultralong-life aqueous zinc-ion hybrid capacitors based on pseudocapacitive charge storage. Nano-Micro Lett. **11**, 94 (2019). https://doi.org/10.1007/s40820-019-0328-3

[S14] J. Wang, J. Polleux, J. Lim, B. Dunn, Pseudocapacitive contributions to electrochemical energy storage in TiO_2 (anatase) nanoparticles. J. Phys. Chem. C **111**(40), 14925-14931 (2007). https://doi.org/10.1021/jp074464w

[S15] C. Li, X. Shi, S. Liang, X. Ma, M. Han et al., Spatially homogeneous copper foam as surface dendrite-free host for zinc metal anode. Chem. Eng. J. **379**, 122248 (2020). https://doi.org/10.1016/j.cej.2019.122248

[S16] Z. Kang, C. Wu, L. Dong, W. Liu, J. Mou et al., 3D porous copper skeleton supported zinc anode toward high capacity and long cycle life zinc ion batteries. ACS Sustainable Chem. Eng. **7**(3), 3364-3371 (2019). https://doi.org/10.1021/acssuschemeng.8b05568

[S17] Y. Tian, Y. An, C. Wei, B. Xi, S. Xiong et al., Flexible and free-standing $Ti_3C_2T_x$ MXene@Zn paper for dendrite-free aqueous zinc metal batteries and nonaqueous lithium metal batteries. ACS Nano **13**(10), 11676-11685 (2019). https://doi.org/10.1021/acsnano.9b05599

[S18] Y. Zeng, X. Zhang, R. Qin, X. Liu, P. Fang et al., Dendrite-free zinc deposition induced by multifunctional CNT frameworks for stable flexible Zn-ion batteries. Adv. Mater. **31**(36), 1903675 (2019). https://doi.org/10.1002/adma.201903675

[S19] R. Yuksel, O. Buyukcakir, W.K. Seong, R.S. Ruoff, Metal-organic framework integrated anodes for aqueous zinc-ion batteries. Adv. Energy Mater. **10**(16), 1904215 (2020). https://doi.org/10.1002/aenm.201904215

[S20] K. Hu, X. Guan, R. Lv, G. Li, Z. Hu et al., Stabilizing zinc metal anodes by artificial solid electrolyte interphase through a surface ion-exchanging strategy. Chem. Eng. J. **396**, 125363 (2020). https://doi.org/10.1016/j.cej.2020.125363

[S21] A. Xia, X. Pu, Y. Tao, H. Liu, Y. Wang, Graphene oxide spontaneous reduction and self-assembly on the zinc metal surface enabling a dendrite-free anode for long-life zinc rechargeable aqueous batteries. Appl. Surf. Sci. **481**, 852-859 (2019). https://doi.org/10.1016/j.apsusc.2019.03.197

[S22] K. Zhao, C. Wang, Y. Yu, M. Yan, Q. Wei et al., Ultrathin surface coating enables stabilized zinc metal anode. Adv. Mater. Interfaces **5**(16), 1800848 (2018). https://doi.org/10.1002/admi.201800848

[S23] H. He, H. Tong, X. Song, X. Song, J. Liu, Highly stable Zn metal anodes enabled by atomic layer deposited Al_2O_3 coating for aqueous zinc-ion batteries. J. Mater. Chem. A **8**(16), 7836-7846 (2020). https://doi.org/10.1039/D0TA00748J

[S24] W. Dong, J.L. Shi, T.S. Wang, Y.X. Yin, C.R. Wang et al., 3D zinc@carbon fiber composite framework anode for aqueous Zn–MnO_2 batteries. RSC Adv. **8**(34), 19157-19163 (2018). https://doi.org/10.1039/C8RA03226B

[S25] A. Wang, W. Zhou, A. Huang, M. Chen, J. Chen et al., Electrodeposition of MnO_2 nanoflakes onto carbon nanotube film towards high-performance flexible quasi-solid-state Zn-MnO_2 batteries. J. Electroanal. Chem. **873**, 114392 (2020). https://doi.org/10.1016/j.jelechem.2020.114392

[S26] L. Ma, Q. Li, Y. Ying, F. Ma, S. Chen et al., Toward practical high-areal-capacity aqueous zinc-metal batteries: quantifying hydrogen evolution and a solid-ion conductor for stable zinc anodes. Adv. Mater. **33**(12), 2007406 (2021). https://doi.org/10.1002/adma.202007406
